# 3,4-Bis[1-(prop-2-yn­yl)-1*H*-indol-3-yl]-1*H*-pyrrole-2,5-dione

**DOI:** 10.1107/S1600536813012889

**Published:** 2013-05-18

**Authors:** Mu-Hua Huang, Yong-Chen Gao, Feng-Ling Yang, Yun-Jun Luo

**Affiliations:** a School of Materials Science and Engineering, Beijing Institute of Technology, Beijing 100081, People’s Republic of China; b Department of Chemistry, Zhengzhou University, Zhengzhou 450052, People’s Republic of China

## Abstract

In the title mol­ecule, C_26_H_17_N_3_O_2_, both indole ring systems are essentially planar, with maximum deviations of 0.019 (2) and 0.033 (1) Å for the N atoms, and form dihedral angles of 34.40 (9) and 45.06 (8)° with the essentially planar pyrrole ring [maximum deviation = 0.020 (2) Å]. The dihedral angle between the two indole ring systems is 58.78 (6)°. In the crystal, mol­ecules are connected by pairs of N—H⋯O hydrogen bonds, forming inversion dimers and generating *R*_2_^2^(8) rings. Weak π–π stacking inter­actions, with a centroid–centroid distance of 3.983 (2) Å, are also observed.

## Related literature

For the importance of bis­indolylmale­imides in medicinal chemistry, see: Bulbule *et al.* (2008 ▶); Wang *et al.* (2012 ▶) and in materials science, see: Chiu *et al.* (2003 ▶); Kaletas *et al.* (2005 ▶); Lin *et al.* (2010 ▶); Nakazono *et al.* (2007 ▶); Yeh *et al.* (2006 ▶). For the isolation of bis­indolylmale­imides from natural products, see: Kamata *et al.* (2006 ▶). For the synthesis of bis­indol­yl­mal­e­­i­mides, see: Prateeptongkum *et al.* (2010 ▶). For a related crystal structure, see: Huang *et al.* (2012 ▶). For hydrogen-bond graph-set motifs, see: Bernstein *et al.* (1995 ▶).
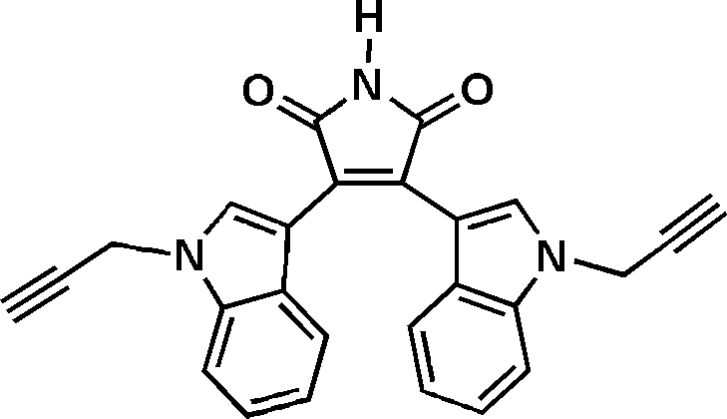


## Experimental

### 

#### Crystal data

C_26_H_17_N_3_O_2_
*M* *_r_* = 403.43
Triclinic, 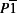

*a* = 8.8015 (14) Å
*b* = 11.2619 (14) Å
*c* = 11.838 (3) Å
α = 62.860 (17)°
β = 73.625 (16)°
γ = 79.593 (12)°
*V* = 999.9 (3) Å^3^
*Z* = 2
Mo *K*α radiation
μ = 0.09 mm^−1^
*T* = 102 K
0.11 × 0.10 × 0.07 mm

#### Data collection

Bruker SMART CCD diffractometer
Absorption correction: multi-scan (*SADABS*; Bruker, 2002 ▶) *T*_min_ = 0.991, *T*_max_ = 0.994
6379 measured reflections
3920 independent reflections
3113 reflections with *I* > 2σ(*I*)
*R*_int_ = 0.024

#### Refinement

*R*[*F* ^2^ > 2σ(*F* ^2^)] = 0.049
*wR*(*F* ^2^) = 0.114
*S* = 1.03
3920 reflections
280 parameters
H-atom parameters constrained
Δρ_max_ = 0.37 e Å^−3^
Δρ_min_ = −0.23 e Å^−3^

### 

Data collection: *SMART* (Bruker, 2007 ▶); cell refinement: *SAINT* (Bruker, 2007 ▶); data reduction: *SAINT*; program(s) used to solve structure: *SHELXS97* (Sheldrick, 2008 ▶); program(s) used to refine structure: *SHELXL97* (Sheldrick, 2008 ▶); molecular graphics: *PLATON* (Spek, 2009 ▶) and *DIAMOND* (Brandenburg, 2006 ▶); software used to prepare material for publication: *SHELXTL* (Sheldrick, 2008 ▶).

## Supplementary Material

Crystal structure: contains datablock(s) I, global. DOI: 10.1107/S1600536813012889/lh5613sup1.cif

Structure factors: contains datablock(s) I. DOI: 10.1107/S1600536813012889/lh5613Isup2.hkl

Supplementary material file. DOI: 10.1107/S1600536813012889/lh5613Isup3.cml

Additional supplementary materials:  crystallographic information; 3D view; checkCIF report

## Figures and Tables

**Table 1:** Hydrogen-bond geometry (Å, °)

*D*—H⋯*A*	*D*—H	H⋯*A*	*D*⋯*A*	*D*—H⋯*A*
N1—H1⋯O1^i^	0.88	2.01	2.872 (2)	165

Symmetry code: (i) 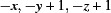.

**Fig. 1.**
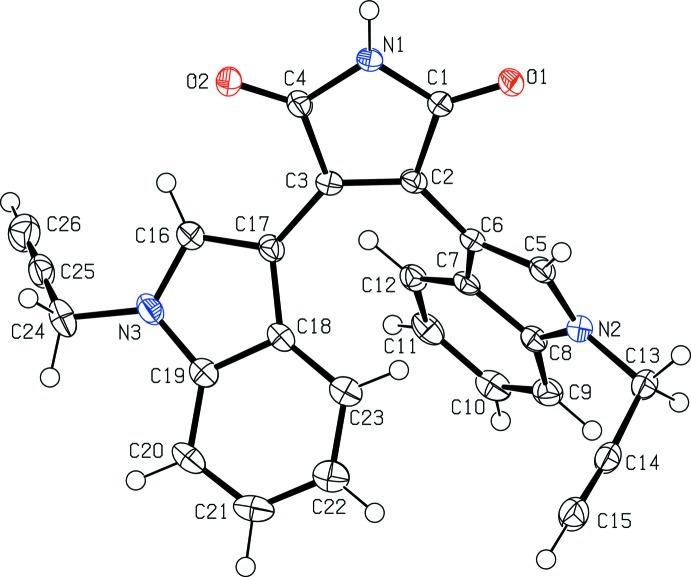
The molecular structure of the title compound with displacement ellipsoids drawn at the 50% probability level.

**Fig. 2.**
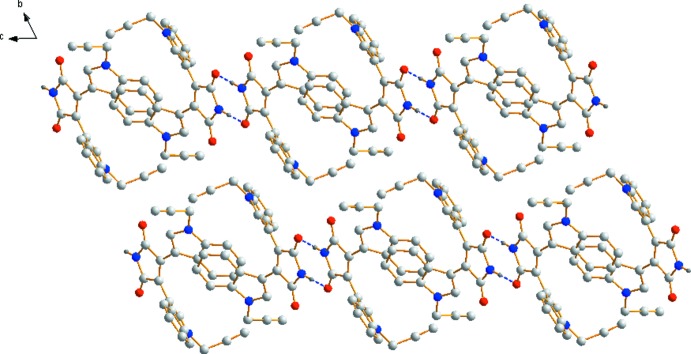
A portion of the crystal packing viewed approximately along the a axis. The dashed lines indicate N—H···O hydrogen bonds.

## supplementary crystallographic information

### Comment

Bisindolylmaleimides are important in medicinal chemistry (Bulbule *et al.*, 2008; Wang *et al.*, 2012) and in the field of materials science (Chiu *et al.*, 2003; Kaletas *et al.*, 2005; Lin *et al.*, 2010; Nakazono *et al.*, 2007; Yeh *et al.*, 2006). Bisindolylmaleimides have been isolated from natural products (Kamata *et al.*, 2006). The synthesis of bisindolylmaleimides (Prateeptongkum *et al.*, 2010) and an example of a related crystal structure (Huang *et al.*, 2012) have been reported.

The molecular structure of the title compound is shown in Fig. 1. Both indole ring systems are essentially planar with maximum deviations of 0.019 (2)Å for N3 and 0.033 (1)Å for N2 and these ring systems form dihedral angles of 34.40 (9)Å [N3/C16-C23] and 45.06 (8)Å [N2/C5-C12] with the essentially planar pyrrole ring [N1/C1-C4] (maximum deviation 0.020 (2)Å for C1). The dihedral angle between the two indole ring systems is 58.78 (6)°. In the crystal, molecules are connected by pairs of N—H···O hydrogen bonds to form inversion dimers (Fig. 2) generating R^2^_2_(8) rings (Bernstein *et al.*, 1995). Weak π–π stacking interactions, with a Cg···Cg(2-x, 1-y, -z) distance of 3.983 (2)Å, are also observed [Cg is the centroid of the C18-C23 ring].

### Experimental

The title compound was prepared by *N*-alkylation of 3, 4-di(1*H*-indol-3-yl)-1*H*-pyrrole-2,5-dione by propargyl bromide with the aid of NaH freshly distilled THF under N_2_ atmosphere. The reaction was initiated at 273K for 5 h. The reaction was quenched with sat. NH_4_Cl at 273K, extracted with EtOAc, dried over anhydrous Na_2_SO_4_ and concentrated *in vacuo*. The residue was purified by f.c.c.(silica gel, eluted with 14% EtOAc in Petroleum Ether) to give the title compound in a yield of 83%, which provided the sample suitable for X-ray analysis after natural evaporation of solvents.

### Refinement

All H atoms were palced in calculated positions with C—H = 0.95Å (aromatic and acetylene hydrogens), 0.99Å (methylene) and N—H = 0.88 Å. They were refined in a riding-model approximation with U_iso_(H) = 1.2U_eq_(C,N).

### Crystal data

**Table:**

C_26_H_17_N_3_O_2_	*V* = 999.9 (3) Å^3^
*M**_r_* = 403.43	*Z* = 2
Triclinic, *P*1	*F*(000) = 420
Hall symbol: -P 1	*D*_x_ = 1.340 Mg m^−^^3^
*a* = 8.8015 (14) Å	Mo *K*α radiation, λ = 0.71073 Å
*b* = 11.2619 (14) Å	θ = 1.5–51.8°
*c* = 11.838 (3) Å	µ = 0.09 mm^−^^1^
α = 62.860 (17)°	*T* = 102 K
β = 73.625 (16)°	Block, colorless
γ = 79.593 (12)°	0.11 × 0.10 × 0.07 mm

### Data collection

**Table:**

Bruker SMART CCD diffractometer	3920 independent reflections
Radiation source: fine-focus sealed tube	3113 reflections with *I* > 2σ(*I*)
Graphite monochromator	*R*_int_ = 0.024
φ and ω scans	θ_max_ = 26.0°, θ_min_ = 3.3°
Absorption correction: multi-scan (*SADABS*; Bruker, 2002)	*h* = −10→9
*T*_min_ = 0.991, *T*_max_ = 0.994	*k* = −13→13
6379 measured reflections	*l* = −11→14

### Refinement

**Table:**

Refinement on *F*^2^	Primary atom site location: structure-invariant direct methods
Least-squares matrix: full	Secondary atom site location: difference Fourier map
*R*[*F*^2^ > 2σ(*F*^2^)] = 0.049	Hydrogen site location: inferred from neighbouring sites
*wR*(*F*^2^) = 0.114	H-atom parameters constrained
*S* = 1.03	*w* = 1/[σ^2^(*F*_o_^2^) + (0.0438*P*)^2^ + 0.3377*P*] where *P* = (*F*_o_^2^ + 2*F*_c_^2^)/3
3920 reflections	(Δ/σ)_max_ < 0.001
280 parameters	Δρ_max_ = 0.37 e Å^−^^3^
0 restraints	Δρ_min_ = −0.23 e Å^−^^3^

### Special details

**Table:**

Geometry. All e.s.d.'s (except the e.s.d. in the dihedral angle between two l.s. planes) are estimated using the full covariance matrix. The cell e.s.d.'s are taken into account individually in the estimation of e.s.d.'s in distances, angles and torsion angles; correlations between e.s.d.'s in cell parameters are only used when they are defined by crystal symmetry. An approximate (isotropic) treatment of cell e.s.d.'s is used for estimating e.s.d.'s involving l.s. planes.
Refinement. Refinement of *F*^2^ against ALL reflections. The weighted *R*-factor *wR* and goodness of fit *S* are based on *F*^2^, conventional *R*-factors *R* are based on *F*, with *F* set to zero for negative *F*^2^. The threshold expression of *F*^2^ > σ(*F*^2^) is used only for calculating *R*-factors(gt) *etc*. and is not relevant to the choice of reflections for refinement. *R*-factors based on *F*^2^ are statistically about twice as large as those based on *F*, and *R*- factors based on ALL data will be even larger.

### Fractional atomic coordinates and isotropic or equivalent isotropic displacement parameters (Å^2^)

**Table:**

	*x*	*y*	*z*	*U*_iso_*/*U*_eq_	
O2	0.33248 (15)	0.75325 (12)	0.29355 (12)	0.0242 (3)	
O1	0.11940 (15)	0.34749 (12)	0.48806 (13)	0.0248 (3)	
C12	0.7380 (2)	0.27334 (17)	0.39074 (16)	0.0203 (4)	
H12	0.7278	0.3531	0.4017	0.024*	
C20	0.8983 (2)	0.62081 (19)	−0.09736 (18)	0.0259 (4)	
H20	0.9790	0.6819	−0.1502	0.031*	
C19	0.7956 (2)	0.62914 (17)	0.01340 (17)	0.0200 (4)	
N2	0.48798 (18)	0.09433 (14)	0.33954 (14)	0.0202 (3)	
C18	0.6728 (2)	0.54223 (17)	0.09277 (17)	0.0185 (4)	
C23	0.6555 (2)	0.44154 (18)	0.05835 (18)	0.0239 (4)	
H23	0.5741	0.3807	0.1093	0.029*	
N3	0.79109 (19)	0.71857 (15)	0.06452 (15)	0.0253 (4)	
C17	0.5904 (2)	0.58517 (17)	0.19368 (17)	0.0205 (4)	
C7	0.6120 (2)	0.23464 (17)	0.36673 (16)	0.0180 (4)	
C9	0.7717 (2)	0.03492 (18)	0.35768 (18)	0.0245 (4)	
H9	0.7834	−0.0443	0.3456	0.029*	
C14	0.5187 (2)	0.04567 (18)	0.15627 (19)	0.0250 (4)	
C16	0.6670 (2)	0.69296 (18)	0.17015 (19)	0.0260 (4)	
H16	0.6375	0.7427	0.2206	0.031*	
C13	0.4647 (2)	−0.00393 (18)	0.29867 (18)	0.0247 (4)	
H13A	0.3508	−0.0218	0.3255	0.030*	
H13B	0.5249	−0.0889	0.3418	0.030*	
C8	0.6308 (2)	0.11411 (17)	0.35377 (16)	0.0194 (4)	
C4	0.3246 (2)	0.63429 (17)	0.32913 (17)	0.0201 (4)	
C22	0.7586 (2)	0.4323 (2)	−0.05068 (19)	0.0287 (5)	
H22	0.7475	0.3643	−0.0737	0.034*	
C11	0.8770 (2)	0.19342 (19)	0.39814 (17)	0.0250 (4)	
H11	0.9624	0.2179	0.4158	0.030*	
C5	0.3823 (2)	0.19972 (17)	0.34038 (17)	0.0201 (4)	
H5	0.2763	0.2098	0.3312	0.024*	
N1	0.19290 (18)	0.56262 (14)	0.41135 (15)	0.0228 (4)	
H1	0.1059	0.5957	0.4484	0.027*	
C10	0.8942 (2)	0.07670 (19)	0.37999 (18)	0.0271 (4)	
H10	0.9923	0.0251	0.3831	0.033*	
C2	0.3827 (2)	0.41642 (17)	0.35429 (17)	0.0178 (4)	
C15	0.5634 (3)	0.09309 (19)	0.0417 (2)	0.0320 (5)	
H15	0.5993	0.1313	−0.0505	0.038*	
C6	0.4527 (2)	0.28858 (17)	0.35655 (16)	0.0181 (4)	
C3	0.4463 (2)	0.53771 (17)	0.29249 (17)	0.0189 (4)	
C21	0.8777 (2)	0.5207 (2)	−0.12664 (18)	0.0291 (5)	
H21	0.9466	0.5118	−0.2006	0.035*	
C25	0.9581 (2)	0.83373 (18)	0.1088 (2)	0.0277 (5)	
C1	0.2178 (2)	0.43211 (17)	0.42665 (17)	0.0198 (4)	
C24	0.8937 (3)	0.8296 (2)	0.0096 (2)	0.0328 (5)	
H24A	0.8322	0.9148	−0.0306	0.039*	
H24B	0.9822	0.8204	−0.0598	0.039*	
C26	1.0093 (3)	0.83789 (19)	0.1887 (2)	0.0344 (5)	
H26	1.0506	0.8413	0.2531	0.041*	

### Atomic displacement parameters (Å^2^)

**Table:**

	*U*^11^	*U*^22^	*U*^33^	*U*^12^	*U*^13^	*U*^23^
O2	0.0229 (8)	0.0190 (7)	0.0286 (7)	−0.0038 (5)	−0.0003 (6)	−0.0108 (6)
O1	0.0188 (7)	0.0217 (7)	0.0301 (7)	−0.0056 (5)	0.0049 (6)	−0.0124 (6)
C12	0.0228 (10)	0.0222 (9)	0.0144 (8)	−0.0046 (7)	−0.0031 (7)	−0.0062 (8)
C20	0.0177 (10)	0.0301 (11)	0.0183 (9)	−0.0005 (8)	−0.0007 (8)	−0.0027 (9)
C19	0.0159 (10)	0.0201 (9)	0.0194 (9)	0.0026 (7)	−0.0061 (7)	−0.0046 (8)
N2	0.0214 (9)	0.0190 (8)	0.0198 (8)	−0.0028 (6)	−0.0025 (6)	−0.0088 (7)
C18	0.0136 (9)	0.0201 (9)	0.0162 (9)	0.0001 (7)	−0.0044 (7)	−0.0029 (8)
C23	0.0204 (10)	0.0291 (10)	0.0203 (9)	−0.0001 (8)	−0.0058 (8)	−0.0088 (8)
N3	0.0210 (9)	0.0246 (9)	0.0238 (8)	−0.0074 (6)	0.0013 (7)	−0.0065 (7)
C17	0.0157 (10)	0.0184 (9)	0.0221 (9)	−0.0007 (7)	−0.0010 (7)	−0.0064 (8)
C7	0.0184 (10)	0.0197 (9)	0.0114 (8)	−0.0020 (7)	−0.0003 (7)	−0.0043 (7)
C9	0.0269 (11)	0.0213 (10)	0.0193 (9)	0.0018 (8)	−0.0027 (8)	−0.0063 (8)
C14	0.0304 (11)	0.0198 (10)	0.0279 (11)	−0.0038 (8)	−0.0065 (9)	−0.0120 (9)
C16	0.0244 (11)	0.0236 (10)	0.0263 (10)	−0.0048 (8)	0.0023 (8)	−0.0110 (9)
C13	0.0310 (12)	0.0204 (10)	0.0247 (10)	−0.0053 (8)	−0.0044 (9)	−0.0110 (8)
C8	0.0213 (10)	0.0196 (9)	0.0138 (8)	−0.0031 (7)	−0.0021 (7)	−0.0045 (7)
C4	0.0183 (10)	0.0212 (10)	0.0195 (9)	−0.0049 (7)	−0.0012 (7)	−0.0082 (8)
C22	0.0279 (12)	0.0329 (11)	0.0241 (10)	0.0027 (8)	−0.0087 (9)	−0.0112 (9)
C11	0.0198 (10)	0.0327 (11)	0.0170 (9)	−0.0050 (8)	−0.0048 (8)	−0.0043 (8)
C5	0.0166 (10)	0.0211 (9)	0.0187 (9)	−0.0035 (7)	0.0005 (7)	−0.0070 (8)
N1	0.0174 (8)	0.0203 (8)	0.0274 (8)	−0.0028 (6)	0.0058 (7)	−0.0133 (7)
C10	0.0211 (11)	0.0293 (11)	0.0211 (10)	0.0048 (8)	−0.0042 (8)	−0.0052 (9)
C2	0.0149 (9)	0.0219 (9)	0.0171 (9)	−0.0024 (7)	−0.0017 (7)	−0.0093 (8)
C15	0.0466 (14)	0.0262 (11)	0.0252 (11)	−0.0083 (9)	−0.0066 (10)	−0.0114 (9)
C6	0.0178 (10)	0.0188 (9)	0.0147 (8)	−0.0031 (7)	0.0013 (7)	−0.0069 (7)
C3	0.0172 (10)	0.0205 (9)	0.0185 (9)	−0.0009 (7)	−0.0015 (7)	−0.0094 (8)
C21	0.0249 (11)	0.0393 (12)	0.0197 (10)	0.0076 (9)	−0.0048 (8)	−0.0135 (9)
C25	0.0206 (11)	0.0220 (10)	0.0340 (11)	−0.0060 (8)	0.0004 (9)	−0.0088 (9)
C1	0.0190 (10)	0.0199 (9)	0.0201 (9)	−0.0036 (7)	−0.0004 (8)	−0.0099 (8)
C24	0.0291 (12)	0.0299 (11)	0.0305 (11)	−0.0137 (9)	0.0008 (9)	−0.0055 (9)
C26	0.0323 (13)	0.0262 (11)	0.0406 (13)	−0.0017 (9)	−0.0068 (10)	−0.0118 (10)

### Geometric parameters (Å, º)

**Table:**

O2—C4	1.216 (2)	C14—C15	1.180 (3)
O1—C1	1.224 (2)	C14—C13	1.471 (3)
C12—C11	1.380 (3)	C16—H16	0.9500
C12—C7	1.402 (2)	C13—H13A	0.9900
C12—H12	0.9500	C13—H13B	0.9900
C20—C21	1.375 (3)	C4—N1	1.388 (2)
C20—C19	1.399 (3)	C4—C3	1.506 (2)
C20—H20	0.9500	C22—C21	1.388 (3)
C19—N3	1.382 (2)	C22—H22	0.9500
C19—C18	1.407 (2)	C11—C10	1.402 (3)
N2—C5	1.371 (2)	C11—H11	0.9500
N2—C8	1.383 (2)	C5—C6	1.374 (2)
N2—C13	1.459 (2)	C5—H5	0.9500
C18—C23	1.412 (3)	N1—C1	1.380 (2)
C18—C17	1.449 (3)	N1—H1	0.8800
C23—C22	1.389 (3)	C10—H10	0.9500
C23—H23	0.9500	C2—C3	1.359 (2)
N3—C16	1.362 (2)	C2—C6	1.451 (2)
N3—C24	1.462 (2)	C2—C1	1.489 (2)
C17—C16	1.374 (2)	C15—H15	0.9500
C17—C3	1.449 (2)	C21—H21	0.9500
C7—C8	1.410 (2)	C25—C26	1.178 (3)
C7—C6	1.439 (2)	C25—C24	1.463 (3)
C9—C10	1.382 (3)	C24—H24A	0.9900
C9—C8	1.390 (3)	C24—H24B	0.9900
C9—H9	0.9500	C26—H26	0.9500
			
C11—C12—C7	118.76 (17)	O2—C4—N1	124.49 (17)
C11—C12—H12	120.6	O2—C4—C3	128.73 (16)
C7—C12—H12	120.6	N1—C4—C3	106.75 (14)
C21—C20—C19	117.34 (18)	C21—C22—C23	121.13 (19)
C21—C20—H20	121.3	C21—C22—H22	119.4
C19—C20—H20	121.3	C23—C22—H22	119.4
N3—C19—C20	129.06 (17)	C12—C11—C10	121.12 (18)
N3—C19—C18	107.88 (16)	C12—C11—H11	119.4
C20—C19—C18	123.06 (17)	C10—C11—H11	119.4
C5—N2—C8	109.07 (14)	N2—C5—C6	109.78 (16)
C5—N2—C13	124.26 (16)	N2—C5—H5	125.1
C8—N2—C13	125.14 (15)	C6—C5—H5	125.1
C19—C18—C23	117.57 (16)	C1—N1—C4	110.35 (15)
C19—C18—C17	106.66 (15)	C1—N1—H1	124.8
C23—C18—C17	135.75 (17)	C4—N1—H1	124.8
C22—C23—C18	119.34 (18)	C9—C10—C11	121.56 (18)
C22—C23—H23	120.3	C9—C10—H10	119.2
C18—C23—H23	120.3	C11—C10—H10	119.2
C16—N3—C19	108.84 (15)	C3—C2—C6	129.71 (17)
C16—N3—C24	124.81 (16)	C3—C2—C1	108.11 (15)
C19—N3—C24	126.19 (16)	C6—C2—C1	122.18 (15)
C16—C17—C18	106.03 (16)	C14—C15—H15	180.0
C16—C17—C3	124.76 (17)	C5—C6—C7	106.70 (15)
C18—C17—C3	128.89 (16)	C5—C6—C2	126.26 (17)
C12—C7—C8	118.80 (17)	C7—C6—C2	126.90 (15)
C12—C7—C6	134.27 (16)	C2—C3—C17	131.66 (16)
C8—C7—C6	106.86 (15)	C2—C3—C4	107.35 (15)
C10—C9—C8	116.94 (17)	C17—C3—C4	120.44 (15)
C10—C9—H9	121.5	C20—C21—C22	121.54 (19)
C8—C9—H9	121.5	C20—C21—H21	119.2
C15—C14—C13	175.8 (2)	C22—C21—H21	119.2
N3—C16—C17	110.56 (17)	C26—C25—C24	179.5 (2)
N3—C16—H16	124.7	O1—C1—N1	125.03 (17)
C17—C16—H16	124.7	O1—C1—C2	127.66 (16)
N2—C13—C14	110.29 (15)	N1—C1—C2	107.30 (14)
N2—C13—H13A	109.6	N3—C24—C25	111.76 (16)
C14—C13—H13A	109.6	N3—C24—H24A	109.3
N2—C13—H13B	109.6	C25—C24—H24A	109.3
C14—C13—H13B	109.6	N3—C24—H24B	109.3
H13A—C13—H13B	108.1	C25—C24—H24B	109.3
N2—C8—C9	129.68 (17)	H24A—C24—H24B	107.9
N2—C8—C7	107.57 (16)	C25—C26—H26	180.0
C9—C8—C7	122.74 (17)		
			
C21—C20—C19—N3	179.63 (18)	C13—N2—C5—C6	−167.15 (15)
C21—C20—C19—C18	−1.3 (3)	O2—C4—N1—C1	176.33 (18)
N3—C19—C18—C23	−179.89 (15)	C3—C4—N1—C1	−1.9 (2)
C20—C19—C18—C23	0.8 (3)	C8—C9—C10—C11	0.1 (3)
N3—C19—C18—C17	1.25 (19)	C12—C11—C10—C9	−1.8 (3)
C20—C19—C18—C17	−178.03 (16)	N2—C5—C6—C7	−0.17 (19)
C19—C18—C23—C22	0.0 (3)	N2—C5—C6—C2	175.86 (16)
C17—C18—C23—C22	178.43 (19)	C12—C7—C6—C5	−175.92 (18)
C20—C19—N3—C16	177.35 (18)	C8—C7—C6—C5	0.94 (19)
C18—C19—N3—C16	−1.9 (2)	C12—C7—C6—C2	8.1 (3)
C20—C19—N3—C24	1.8 (3)	C8—C7—C6—C2	−175.05 (16)
C18—C19—N3—C24	−177.39 (17)	C3—C2—C6—C5	−134.3 (2)
C19—C18—C17—C16	−0.2 (2)	C1—C2—C6—C5	46.1 (3)
C23—C18—C17—C16	−178.7 (2)	C3—C2—C6—C7	40.9 (3)
C19—C18—C17—C3	173.55 (18)	C1—C2—C6—C7	−138.68 (18)
C23—C18—C17—C3	−5.0 (3)	C6—C2—C3—C17	11.6 (3)
C11—C12—C7—C8	1.3 (2)	C1—C2—C3—C17	−168.76 (19)
C11—C12—C7—C6	177.89 (18)	C6—C2—C3—C4	−177.07 (17)
C19—N3—C16—C17	1.8 (2)	C1—C2—C3—C4	2.5 (2)
C24—N3—C16—C17	177.39 (17)	C16—C17—C3—C2	−157.5 (2)
C18—C17—C16—N3	−1.0 (2)	C18—C17—C3—C2	29.8 (3)
C3—C17—C16—N3	−175.05 (17)	C16—C17—C3—C4	32.1 (3)
C5—N2—C13—C14	84.9 (2)	C18—C17—C3—C4	−140.54 (18)
C8—N2—C13—C14	−79.4 (2)	O2—C4—C3—C2	−178.66 (18)
C5—N2—C8—C9	−179.60 (18)	N1—C4—C3—C2	−0.5 (2)
C13—N2—C8—C9	−13.3 (3)	O2—C4—C3—C17	−6.2 (3)
C5—N2—C8—C7	1.29 (19)	N1—C4—C3—C17	171.94 (16)
C13—N2—C8—C7	167.59 (15)	C19—C20—C21—C22	0.9 (3)
C10—C9—C8—N2	−176.65 (17)	C23—C22—C21—C20	−0.1 (3)
C10—C9—C8—C7	2.4 (3)	C4—N1—C1—O1	−175.96 (18)
C12—C7—C8—N2	176.07 (15)	C4—N1—C1—C2	3.4 (2)
C6—C7—C8—N2	−1.36 (19)	C3—C2—C1—O1	175.62 (18)
C12—C7—C8—C9	−3.1 (3)	C6—C2—C1—O1	−4.7 (3)
C6—C7—C8—C9	179.45 (16)	C3—C2—C1—N1	−3.8 (2)
C18—C23—C22—C21	−0.3 (3)	C6—C2—C1—N1	175.90 (16)
C7—C12—C11—C10	1.1 (3)	C16—N3—C24—C25	52.3 (3)
C8—N2—C5—C6	−0.7 (2)	C19—N3—C24—C25	−132.89 (19)

### Hydrogen-bond geometry (Å, º)

**Table:**

*D*—H···*A*	*D*—H	H···*A*	*D*···*A*	*D*—H···*A*
N1—H1···O1^i^	0.88	2.01	2.872 (2)	165

Symmetry code: (i) −*x*, −*y*+1, −*z*+1.
